# Mitochondrial dysfunction in aortic aneurysm and dissection: mechanisms and therapeutic implications

**DOI:** 10.3389/fphar.2026.1942911

**Published:** 2026-09-10

**Authors:** Yang Yang, Zhili He, Yan Wang, Shuang Zhao, Tianyu Song

**Affiliations:** 1 School of Medicine, Nanjing University of Chinese Medicine, Nanjing, China; 2 Key Laboratory of Drug Targets and Translational Medicine for Cardio-cerebrovascular Diseases, Medical Basic Research Innovation Center for Cardiovascular and Cerebrovascular Diseases, Ministry of Education, School of Pharmacy, Nanjing Medical University, Nanjing, China

**Keywords:** aortic aneurysm and dissection, mitochondrial biogenesis, mitochondrial dynamics, mitochondrial dysfunction, mitochondrial energy metabolism, oxidative stress

## Abstract

Aortic aneurysm and dissection (AAD) are life-threatening vascular diseases associated with progressive aortic wall degeneration and a high risk of rupture. Despite advances in surgical and endovascular techniques, effective disease-modifying pharmacological therapies remain limited. Mitochondria are central regulators of cellular energy metabolism, redox homeostasis, and apoptosis, and their functional integrity is essential for maintaining vascular homeostasis and aortic wall stability. Increasing evidence indicates that mitochondrial dysfunction plays a pivotal role in the initiation and progression of AAD. In this review, we summarize the major mechanisms by which mitochondrial abnormalities contribute to AAD pathogenesis, including impaired mitochondrial energy metabolism, defective mitochondrial biogenesis, excessive mitochondrial oxidative stress, and imbalanced mitochondrial dynamics. These interconnected processes promote vascular smooth muscle cell (VSMC) phenotypic switching, apoptosis, and senescence, alongside extracellular matrix degradation and inflammatory activation, ultimately culminating in the structural weakening of the aortic wall. We also discuss emerging mitochondria-targeted therapeutic strategies, including interventions aimed at restoring metabolic homeostasis, enhancing mitochondrial biogenesis, suppressing oxidative stress, and regulating mitochondrial fission-fusion balance. Although the clinical translation of these approaches remains hindered by inadequate target specificity and potential off-target effects, ameliorating mitochondrial dysfunction represents a promising strategy for developing non-surgical treatments for AAD. A deeper understanding of mitochondrial regulatory networks may provide new mechanistic insights and therapeutic opportunities for preventing AAD progression.

## Introduction: overview of aortic aneurysm and dissection

Aortic aneurysm and dissection (AAD) encompass a group of severe, life-threatening cardiovascular diseases associated with substantial morbidity and mortality (Liu et al., 2026). The development of aortic aneurysm (AA) involves gradual changes in extracellular matrix (ECM) organization, vascular cell function, and wall integrity, which may ultimately predispose patients to dissection or rupture. AA is generally classified into abdominal aortic aneurysm (AAA) and thoracic aortic aneurysm (TAA). Although both conditions involve pathological remodeling of the aortic wall, they differ in genetic contribution, associated risk factors, and dominant cellular mechanisms (Lu et al., 2021). Thus, although AAA and TAA share several pathological features, their initiating factors and dominant remodeling mechanisms are not identical.

### AAA

AAA is characterized by progressive remodeling of the abdominal aortic wall, accompanied by loss of structural stability and gradual vessel enlargement. Atherosclerotic changes are frequently observed in affected arteries, although the contribution of individual risk factors varies among patients. Smoking, aging, male sex, hypertension, dyslipidemia, and metabolic disorders have all been associated with increased AAA susceptibility (Pinard et al., 2019). Although AAA occurs predominantly in elderly men, women represent a higher proportion of rupture-related cases, suggesting that biological sex may influence disease progression and clinical outcomes (Lo and Schermerhorn, 2016). From a clinical perspective, AAA is usually identified when the abdominal aortic diameter reaches 3.0 cm or greater (Isselbacher et al., 2022). Continued degradation of the aortic wall promotes aneurysmal expansion and increases the risk of rupture. Despite improvements in screening and management, AAA remains a significant cause of cardiovascular mortality, with approximately 4,500 deaths reported annually in the United States (Haque and Bhargava, 2022). Because early AAA often develops without obvious symptoms, diagnosis is frequently delayed until advanced enlargement or rupture occurs. At the tissue level, AAA progression involves coordinated changes in vascular cells and the extracellular environment, including inflammatory activation, ECM remodeling, and alterations in vascular smooth muscle cell (VSMC) phenotype, which collectively weaken aortic wall integrity (Zheng et al., 2024).

### TAA

TAA has a stronger genetic basis than AAA, especially in patients with inherited connective tissue disorders (Ardahanlı. et al., 2025b). Pathogenic variants have been identified in a subset of TAA cases (Pinard et al., 2019). Fibrillin 1 (FBN1) mutations are a major cause of Marfan syndrome (MFS) (Pollock et al., 2021), whereas alterations in TGFBR1 or TGFBR2 are frequently associated with Loeys-Dietz syndrome (LDS) (Calderon-Martinez et al., 2025). Genetic TAA is mainly characterized by degeneration of the aortic media. Disruption of ECM organization and impaired VSMC maintenance weaken the medial layer and reduce aortic wall stability. These changes involve matrix degradation, VSMC loss, and structural disorganization of the media. Unlike AAA, medial degeneration in TAA often occurs without marked atherosclerotic lesions, indicating different pathogenic processes between thoracic and abdominal aortic diseases (Ladich et al., 2016).

### Aortic dissection

Aortic dissection (AD) is characterized by disruption of the aortic wall structure with separation of the medial layer and formation of a false lumen. Although the incidence of AD increases with age, genetically predisposed individuals may develop the disease at younger ages (Parve et al., 2017). An intimal tear is often considered the initiating event, but subsequent progression involves complex remodeling of the aortic wall, including ECM degradation, inflammatory activation, and vascular cell dysfunction (Nienaber et al., 2016). According to the Stanford classification, AD is categorized as type A or type B based on whether the ascending aorta is involved (Nienaber et al., 2016). Hypertension, aging, and metabolic abnormalities are major acquired risk factors. In addition, patients with hereditary thoracic aortic diseases, including MFS and LDS, show increased susceptibility because of impaired structural integrity and mechanical properties of the aortic wall (Calderon-Martinez et al., 2025). Current management of AD mainly relies on surgical repair and endovascular intervention. Pharmacological treatment is primarily used to reduce hemodynamic stress and limit further vascular injury, but therapies that directly modify aortic wall degeneration remain unavailable (Juraszek et al., 2022).

### Cellular mechanisms in the pathogenesis of AAD

Although VSMCs are the predominant cellular component of the aortic media, AAD progression involves extensive interactions among vascular and immune cells within the aortic wall. Endothelial cells (ECs), fibroblasts, and immune populations influence disease evolution through changes in inflammatory signaling, ECM remodeling, and vascular homeostasis. These cellular interactions create a pathological environment that favors progressive aortic wall deterioration.

The tunica intima, composed of a single EC layer resting on a basal lamina, mediates substance exchange and initiates vascular injury responses. The integrity of the endothelial barrier depends on specialized junctional structures, including tight junctions, adherens junctions, and focal adhesions (Teng et al., 2025). Multiple studies demonstrate the critical involvement of endothelial barrier damage in the development of AAA, TAA, and AD, where impaired integrity promotes inflammatory cell infiltration and edema within the aortic wall (Gould et al., 2019; Yang K. et al., 2023; Yang X. et al., 2023). The transition of ECs toward senescent or mesenchymal phenotypes provides substantial mechanistic evidence elucidating the pathogenesis of TAA and AD (Chen Y. et al., 2025; Cao et al., 2026). EC dysfunction in AAD is also characterized by impaired efferocytosis, enhanced endoplasmic reticulum stress, and increased mitochondrial reactive oxygen species (ROS) levels (Luo et al., 2023; Liu et al., 2025; Shao et al., 2026; Zhang W. et al., 2026). Consequently, these dysfunctional ECs induce downstream effects, including platelet and leukocyte activation, VSMC phenotypic alteration, and ECM remodeling (Song et al., 2026).

The tunica media consists primarily of VSMCs residing within a highly organized ECM enriched with elastin and collagen fibers. VSMCs maintain aortic wall function by regulating vascular contraction and continuously adapting ECM organization to mechanical demands. During AAD development, however, VSMCs undergo a phenotypic switching from a contractile state toward a synthetic phenotype, accompanied by increased proliferative activity, migration, and changes in ECM regulation. This process may initially represent an adaptive response to vascular injury, but persistent activation contributes to excessive vascular remodeling and progressive loss of aortic wall integrity (Jarad et al., 2025). The contribution of VSMC dysfunction to hereditary aortic disease is also supported by genetic studies, in which mutations affecting contractile genes such as *ACTA2* and *MYH11* have been associated with increased susceptibility to familial TAA and AD (Pan et al., 2022; Liu et al., 2024).

The tunica adventitia is not merely a structural layer surrounding the aorta but also contributes to the pathological remodeling process during AAD. Adventitial fibroblasts regulate ECM turnover and interact with neighboring vascular cells and immune components, thereby influencing the local microenvironment of the aortic wall (Poduri et al., 2015; Chen et al., 2022). During AAD progression, fibroblast activation may disturb ECM homeostasis and enhance inflammatory signaling within the adventitia. These alterations can facilitate immune cell recruitment and further impair the structural integrity of the vascular wall (Mohanta et al., 2025). In human AD specimens, increased B-cell accumulation has been observed compared with non-diseased tissues. Although the precise role of B cells in AD remains unclear, activated B cells may participate in local inflammatory regulation through antibody production and cytokine secretion, which may contribute to ECM remodeling and vascular inflammation (Hou et al., 2022). Together, alterations in adventitial fibroblasts and immune cell composition may represent important components of the inflammatory remodeling process underlying AAD.

## Molecular hallmarks of mitochondrial dysfunction in cardiovascular diseases

Within cardiovascular biology, mitochondria do not merely produce adenosine triphosphate (ATP); they also regulate how cells adapt metabolically and respond to stress. While oxidative phosphorylation (OXPHOS) is the primary pathway for ATP generation, broader mitochondrial networks control substrate preferences, including fatty acid oxidation. This metabolic activity is tightly integrated with redox regulation, as OXPHOS directly generates mitochondrial ROS (Li M. et al., 2025). Additionally, these organelles act as signaling hubs. By altering the abundance of messenger molecules like ROS, acetyl-CoA, and calcium, they modulate nuclear transcription to influence cellular processes including proliferation, autophagy, and apoptosis (Weinberg et al., 2015). When mitochondria fail, the resulting cardiovascular dysfunction stems not only from energy depletion but from the breakdown of these vital signaling networks.

### Mitochondrial energy metabolism

Mitochondrial energy metabolism is much more than a simple drop in ATP production; it actively drives cardiovascular disease progression. Healthy cells easily maintain metabolic flexibility, seamlessly switching between carbohydrates, fatty acids, and amino acids to meet fluctuating energy demands. Under chronic metabolic stress, this adaptive cushion wears thin. Crippling the tricarboxylic acid (TCA) cycle-OXPHOS pathway does not just slow down respiration-it strips the cells of their ability to adapt to environmental insults, locking them into a state of metabolic vulnerability.

Defects in mitochondrial respiration are not restricted to a single component of the respiratory chain, but rather reflect impaired metabolic flexibility under cardiovascular stress. Although the affected respiratory complexes vary among different disease settings, reduced electron transport efficiency appears to be a common consequence of mitochondrial dysfunction. NADH: ubiquinone oxidoreductase subunit S4 deficiency, for instance, disrupts complex I activity and decreases mitochondrial respiration, thereby aggravating myocardial injury after infarction (Cai et al., 2023). In cardiomyocytes lacking succinate dehydrogenase complex assembly factor 4, impaired complex II stability is accompanied by reduced respiratory capacity, increased dynamin related protein 1 (DRP1) activation, mitochondrial fragmentation, and mitophagy, which together contribute to cardiomyopathy progression (Wang et al., 2022). Similar metabolic alterations have been observed beyond individual respiratory complexes, as decreased expression of TCA cycle enzymes, including citrate synthase and malate dehydrogenase, occurs in atrial cardiomyocytes from patients with atrial fibrillation (Tu et al., 2014).

Metabolic remodeling in vascular diseases reflects broader alterations in mitochondrial substrate utilization and energy handling. Such remodeling is accompanied by changes in substrate preference and mitochondrial metabolic utilization. In pulmonary arterial hypertension (PAH), pulmonary artery smooth muscle cells show lower OXPHOS activity and greater reliance on glycolysis, together with changes in fatty acid metabolism (Zhuang et al., 2019). Similar metabolic adaptations have been described in VSMCs during vascular remodeling and atherosclerosis, characterized by increased glycolytic activity and altered mitochondrial metabolism (Wall et al., 2018). These findings support the view that mitochondrial dysfunction may influence vascular remodeling through impaired metabolic flexibility, rather than through reduced energy production alone. The mechanisms underlying this metabolic shift are complex, and several upstream regulators have been proposed. In atherosclerotic models, arachidonate 12-lipoxygenase (ALOX12) has been linked to changes in AMP-activated protein kinase (AMPK) signaling, a pathway involved in mitochondrial energy metabolism. Increased ALOX12 activity has been associated with reduced AMPK-related mitochondrial regulation and a shift toward glycolytic metabolism. Conversely, targeting the ALOX12-AMPK axis may provide a potential approach to improve oxidative metabolism, although its relevance in vascular disease requires further validation (Olkowicz et al., 2024).

### Mitochondrial biogenesis

Alterations in mitochondrial biogenetic capacity have been reported in cardiovascular diseases, particularly under conditions of persistent cellular stress. Although mitochondrial biogenesis is commonly viewed as a mechanism for maintaining mitochondrial abundance and function, its regulation involves a broader network rather than a single transcriptional pathway. The peroxisome proliferator-activated receptor gamma coactivator 1-alpha (PGC-1α)-centered regulatory axis, including nuclear respiratory factor 1 (NRF1) and nuclear respiratory factor 2 (NRF2), contributes to transcription factor A, mitochondrial (TFAM) expression and mitochondrial genome maintenance, thereby supporting respiratory capacity (Liu et al., 2023; Zhao et al., 2023). In addition, metabolic signals such as AMPK activation, oxidized and reduced nicotinamide adenine dinucleotide (NAD^+^/NADH)-dependent sirtuin 1 (SIRT1) signaling, and Ca^2+^-responsive pathways can influence this process, indicating that mitochondrial biogenesis is closely linked to the metabolic state of the cell (Hees and Harbauer, 2022).

Impaired mitochondrial biogenesis has been observed in cardiovascular disorders and is often accompanied by reduced expression of mitochondrial regulators, decreased mitochondrial DNA (mtDNA) content, and compromised metabolic capacity. Such defects may limit mitochondrial adaptation under stress and contribute to abnormal ROS production and cellular dysfunction (Chistiakov et al., 2018). Experimental activation of PGC-1α has been shown to enhance mitochondrial gene expression and improve mitochondrial function. In atherosclerosis, reduced PGC-1α expression has been detected in human plaques, particularly in clinically unstable lesions (Sung et al., 2024). Consistently, increasing PGC-1α activity in VSMCs suppresses inflammatory responses, oxidative stress, cellular senescence, and matrix remodeling, thereby limiting atherosclerotic progression (Wei et al., 2021). These findings suggest that defective mitochondrial biogenesis may influence vascular disease not only by reducing mitochondrial capacity but also by altering the inflammatory and remodeling behavior of vascular cells.

### Mitochondrial redox homeostasis

Mitochondrial redox changes actively shape cardiovascular stress responses, exerting influence far beyond simple oxidative damage. Under physiological conditions, low levels of mitochondrial ROS are not waste products; instead, they function as essential messengers in cellular signaling. But balance is everything. When ROS generation overwhelms antioxidant defenses, this homeostatic disruption inflicts molecular damage and warps cellular stress pathways (Peoples et al., 2019). Metabolic disorders showcase this toxic shift; in type 2 diabetes, for instance, runaway mitochondrial ROS accumulation contributes to diabetic cardiomyopathy (Rovira-Llopis et al., 2017). Cardiomyocytes bear the brunt of this imbalance, as excessive ROS alters calcium handling and metabolic networks-critical defects that directly impair contractility during pathological remodeling (Münzel et al., 2015). A similar cascade unfolds in myocardial ischemia-reperfusion injury, where metabolic cripples amplify ROS-dependent damage (Tian et al., 2023). Further complicating this landscape, activating arachidonate 15-lipoxygenase-1 triggers the peroxidation of polyunsaturated fatty acid-containing phospholipids, offering yet another route to mitochondrial and cell death (Ma et al., 2022).

In vascular tissues, oxidative stress is closely linked to endothelial dysfunction and inflammation (Ardahanlı İ. et al., 2025). Excessive ROS production promotes endothelial activation and adhesion molecule expression, facilitating inflammatory cell recruitment and vascular remodeling (Nowak et al., 2017; Negre-Salvayre et al., 2020). In PAH, elevated ROS levels have been associated with pulmonary vascular remodeling, where oxidative stress promotes VSMC proliferation, migration, and inflammatory activation. These findings suggest that mitochondrial ROS are not merely byproducts of vascular injury but may actively participate in regulating vascular cell behavior during disease progression (Nowak et al., 2017).

### Mitochondrial dynamics

Cardiovascular health depends on the constant structural reshaping of mitochondria through fission and fusion. Beyond merely sculpting mitochondrial morphology, this dynamic interplay secures proper organelle organization and quality control. When this balance breaks down, adaptation fails, altering how vascular cells navigate pathological stress. Fission relies heavily on DRP1. To induce fragmentation, DRP1 docks at the outer mitochondrial membrane (OMM), a recruitment drive tightly policed by adaptors like mitochondrial fission 1 protein (FIS1), mitochondrial fission factor (MFF), and mitochondrial dynamics proteins 49 and 51 (MiD49/51) (Li W. et al., 2025). Fusion, by contrast, operates under a different molecular toolkit. Here, mitofusin 1 (MFN1), mitofusin 2 (MFN2), and optic atrophy 1 (OPA1) take charge, coordinating outer membrane fusion and stabilizing cristae architecture (Gao et al., 2017). Ultimately, mitochondrial shape is not the work of a single pathway, but the outcome of this continuous molecular balance.

Disturbed mitochondrial dynamics has been reported in cardiovascular remodeling. In diabetic cardiomyopathy, enhanced mitochondrial fragmentation is accompanied by reduced MFN2 expression, whereas MFN2 restoration partially improves mitochondrial abnormalities and cardiac function (Hu et al., 2019). During ischemia-reperfusion injury, Ca^2+^ overload activates calcineurin signaling and promotes DRP1 Ser637 dephosphorylation, favoring mitochondrial fission (Piao et al., 2024). Alterations in fusion-related proteins, including OPA1 and MFN1/MFN2, have also been linked to mitochondrial fragmentation and cardiac dysfunction (Guo et al., 2018; Ramaccini et al., 2020). These observations indicate that impaired mitochondrial dynamics reflects a broader failure of mitochondrial network regulation rather than simply excessive fission.

Vascular remodeling disorders provide additional evidence linking mitochondrial dynamics with pathological changes. In PAH, increased mitochondrial fission in pulmonary artery smooth muscle cells is associated with disease progression, and mitochondrial fragmentation may influence vascular cell behavior (Chen et al., 2018). DRP1 phosphorylation appears to provide another layer of regulation, because reduced Ser637 phosphorylation and increased Ser616 phosphorylation favor a fission-prone state (Chen et al., 2020). In atherosclerosis, MFN2 downregulation has been linked to VSMC calcification and chondrogenic differentiation, while MFN2 restoration can partially alleviate these changes (Zhang et al., 2022; Zhang et al., 2023).

## Mitochondrial dysfunction as a key contributor to AAD

Mitochondrial dysfunction in AAD is a multifaceted process that spans multiple layers of organelle regulation. At the metabolic core, impaired aconitase 2 (ACO2) activity and sirtuin 3 (SIRT3)-deficiency-driven acetyl-CoA accumulation alter VSMC phenotypes, thereby compromising aortic wall integrity. Concurrently, mitochondrial biogenesis is severely compromised. Dysregulated PGC-1α/TFAM and SIRT6/NFE2L2 signaling pathways leave the cells unable to maintain their mitochondria, pushing VSMC toward senescence and programmed death. This structural decay occurs under a cloud of redox imbalance, where excessive mitochondrial ROS triggers inflammatory cascades and drives matrix remodeling. Compounding these defects, unbalanced dynamics-marked by DRP1-driven fission and failing MFN2/OPA1 fusion-shatter mitochondrial quality control. Ultimately, AAD is driven not by isolated anomalies, but by this multi-front mitochondrial failure.

### Mitochondrial energy metabolism and AAA: From TCA impairment to metabolic reprogramming

In AAA, mitochondrial metabolic alterations have been increasingly linked to VSMC phenotypic switching. Maintenance of the contractile VSMC phenotype depends, at least in part, on mitochondrial ATP production supported by the TCA cycle and OXPHOS (Zhang and Gao, 2021). In angiotensin II (Ang II)-induced AAA models, impaired mitochondrial metabolism is closely associated with diminished ACO2 activity. This enzymatic downregulation disrupts TCA cycle flux and mitochondrial respiration, thereby facilitating VSMC apoptosis (Sun et al., 2022). Mechanistically, ACO2 expression is partly regulated by nuclear receptor subfamily 1 group D member 1 (NR1D1)-dependent transcriptional machinery. Recruitment of the nuclear receptor corepressor 1 (NCOR1)-histone deacetylase 3 corepressor complex by NR1D1 suppresses ACO2 expression and alters mitochondrial metabolic status (Sun et al., 2022). NCOR1 may also affect vascular remodeling through interactions with transcription factors including FOXO3, NFAT5, and ATF3, suggesting that its effects may extend beyond direct regulation of ACO2 and influence contractile gene expression and ECM remodeling during VSMC phenotypic switching (Du et al., 2023). These findings suggest a potential link between altered TCA cycle activity and VSMC remodeling in AAA.

A second aspect of mitochondrial metabolic regulation potentially relevant to AAA involves SIRT3-dependent metabolic homeostasis. SIRT3 regulates mitochondrial metabolism through deacetylation of multiple metabolic proteins and may influence vascular cell metabolism and remodeling. Although direct evidence linking SIRT3 to AAA remains limited, recent studies indicate that SIRT3 deficiency disrupts mitochondrial acetyl-CoA homeostasis, promotes VSMC metabolic reprogramming, and contributes to vascular remodeling and neointimal hyperplasia (You et al., 2025). *In vitro*, SIRT3 deficiency alters TCA cycle activity, OXPHOS function, and acetyl-CoA homeostasis. Platelet-derived growth factor-BB stimulation can further reinforce these metabolic changes, linking mitochondrial dysfunction with VSMC phenotypic switching. Similarly, experimental studies have connected TNF signaling with changes in VSMC mitochondrial respiration and AP-1-dependent transcription (Luo et al., 2024). These alterations are also reflected in changes in ATP production, mitochondrial membrane potential, and NAD^+^/NADH balance (Luo et al., 2024).

Metabolic reprogramming during VSMC phenotypic switching is also evident in the balance between mitochondrial OXPHOS and glycolysis. During AAA development, VSMCs commonly lose contractile characteristics and acquire a more synthetic phenotype, accompanied by a shift from mitochondrial OXPHOS toward glycolytic metabolism (Jia et al., 2022; Rombouts et al., 2022). Evidence from human AAA tissues indicates that pyruvate dehydrogenase kinase 4 (PDK4) expression is increased in AAA. Mechanistic studies have shown that PDK4 promotes metabolic reprogramming in VSMCs, impairs mitochondrial respiration, activates the NLR family pyrin domain containing 3 inflammasome, and induces pyroptosis, thereby exacerbating vascular inflammation and AAA progression (Zhao et al., 2026). Consistent with these findings, VSMC-specific knockout of *Pdk4* reduced AAA formation in male mice (Zhao et al., 2026). Together, these findings provide experimental evidence that PDK4-mediated metabolic remodeling contributes to AAA development.

Epigenetic regulation may further connect glycolytic metabolism with VSMC phenotypic switching in AAA. Histone H4 lysine 16 lactylation (H4K16la) is increased in murine and human AAA tissues. Mechanistic studies in VSMCs and experimental AAA models indicate that H4K16la promotes pyruvate dehydrogenase kinase 1 (PDK1) transcription and thereby enhances glycolytic metabolism in VSMCs. Inhibition of PDK1 disrupts the H4K16la/PDK1/lactate positive-feedback loop, thereby inhibiting VSMC metabolic reprogramming and phenotypic switching (Liu et al., 2026).

### Mitochondrial biogenesis defects and AAD

**FIGURE 1:**
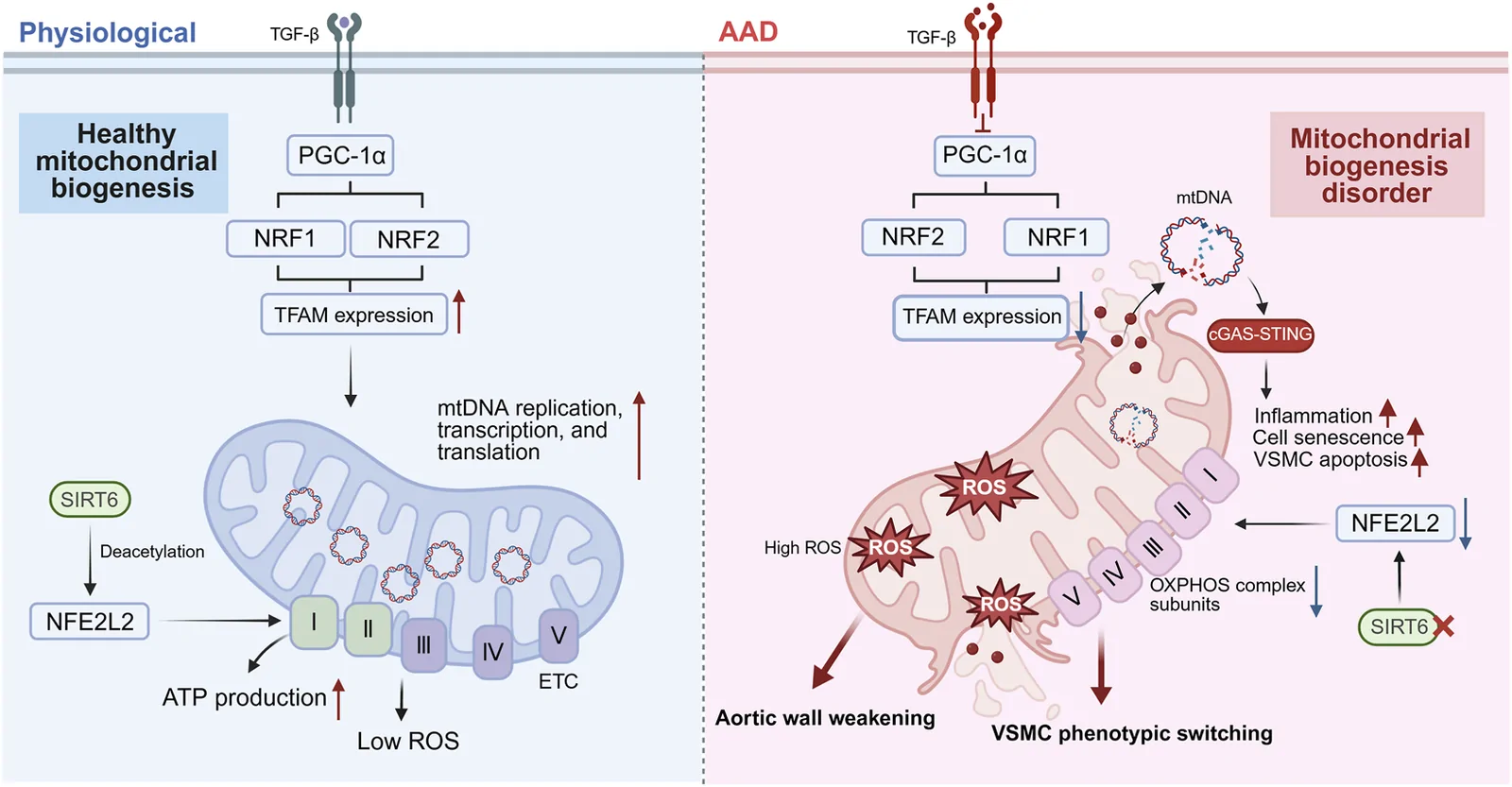
Dysregulation of mitochondrial biogenesis under physiological and AAD pathological conditions. Under physiological conditions, TGF-β signaling and SIRT6 cooperatively regulate mitochondrial biogenesis and homeostasis in VSMCs. TGF-β activates PGC-1α, which regulates NRF1/NRF2 to promote TFAM expression and OXPHOS-related gene transcription. In parallel, SIRT6 enhances NFE2L2 activity via deacetylation. These coordinated processes maintain mtDNA replication, transcription, and translation, ensuring ETC integrity, efficient ATP production, and low ROS levels. In AAD, aberrant TGF-β signaling suppresses PGC-1α activity, while reduced SIRT6 impairs NFE2L2 deacetylation. This leads to decreased TFAM expression and OXPHOS subunits, resulting in impaired mitochondrial biogenesis, mtDNA homeostasis disruption, ETC dysfunction, and excessive ROS accumulation. Damaged mitochondria release mtDNA into the cytoplasm, activating the cGAS-STING pathway. These cellular abnormalities drive local inflammation and senescence, alongside VSMC apoptosis and phenotypic switching. Collectively, these pathological processes compromise the structural integrity of the aortic wall, thereby facilitating the progression of AAD. Created in https://BioRender.com. AAD, aortic aneurysm and dissection; ATP, adenosine triphosphate; ETC, electron transport chain; mtDNA, mitochondrial DNA; NFE2L2, NFE2 like bZIP transcription factor 2; NRF1/NRF2, nuclear respiratory factor 1/2; OXPHOS, oxidative phosphorylation; PGC-1α, peroxisome proliferator-activated receptor gamma coactivator 1-alpha; ROS, reactive oxygen species; SIRT6, sirtuin 6; TFAM, transcription factor A, mitochondrial; TGF-β, transforming growth factor-β and VSMC, vascular smooth muscle cell.

Although mitochondrial biogenesis is disrupted in both TAA and AD, the molecular mechanisms involved appear to differ between the two conditions (Figure 1).

In TAA, mitochondrial biogenesis is mainly associated with dysregulation of the PGC-1α/NRF1/TFAM pathway, particularly in the context of VSMC dysfunction and ECM abnormalities. In Fibulin-4-associated TAA models, ECM abnormalities can enhance TGF-β1 signaling, leading to reduced expression and transcriptional activity of PGC-1α (Yu et al., 2018). As a central regulator of mitochondrial biogenesis, PGC-1α coactivates nuclear respiratory factors such as NRF1, which in turn promotes the expression of downstream mitochondrial genes including TFAM, thereby supporting mtDNA maintenance and mitochondrial biogenesis. Activation of PGC-1α has been shown to reverse mitochondrial dysfunction in Fibulin-4^R/R^ VSMCs and restore their proliferative capacity, suggesting that impaired PGC-1α signaling may contribute to VSMC dysfunction in TAA (van der Pluijm et al., 2018). Further downstream, TFAM is essential for mtDNA maintenance and mitochondrial biogenesis, and its downregulation appears to be associated with TAA progression. FBN1 deficiency has been shown to reduce TFAM expression, which may promote VSMC phenotypic switching and contribute to TAA development (Xia et al., 2023). Consistent with these findings, mitochondrial damage in MFS mice is accompanied by reduced TFAM expression and impaired VSMC contractility, whereas restoration of TFAM expression and mitochondrial metabolic function significantly reduces aneurysm formation (Oller et al., 2021). The consequences of TFAM deficiency may extend beyond impaired mitochondrial biogenesis. Reduced TFAM-mediated maintenance of mtDNA may promote mtDNA instability and cytosolic mtDNA accumulation, which can activate the cGAS-STING pathway and potentially contribute to inflammatory responses during TAA progression (West et al., 2015; Luo et al., 2020). Importantly, evidence from human MFS aortic tissues further supports the relevance of mitochondrial abnormalities to human aortic disease, as reduced expression of mitochondrial respiratory complex subunits and genes involved in mitochondrial biogenesis has been observed in aortic tissues from patients with MFS (Oller et al., 2021).

In AD, mitochondrial biogenesis appears to be regulated through a distinct SIRT6/NFE2L2-dependent mechanism. Sirtuin 6 (SIRT6) has been implicated in maintaining mitochondrial homeostasis in VSMCs through its deacetylase activity. Reduced SIRT6 expression has been detected in human AD tissues, while genetic deletion of SIRT6 increases susceptibility to Ang II-induced AD in mice (Ding Y. N. et al., 2023). Mechanistically, SIRT6 regulates NFE2 like bZIP transcription factor 2 (NFE2L2) signaling and the expression of genes encoding mitochondrial respiratory complex proteins. Disruption of this regulatory relationship may impair mitochondrial biogenesis and mitochondrial homeostasis. These mitochondrial abnormalities are accompanied by enhanced VSMC senescence and apoptosis, which may further compromise aortic wall integrity and increase susceptibility to AD development (Yu et al., 2026). Thus, the SIRT6/NFE2L2 axis may provide a link between mitochondrial homeostasis and VSMC survival in AD. Notably, the reduced SIRT6 expression observed in human AD tissues supports the relevance of this pathway to human disease.

### Mitochondrial oxidative stress and AAD

**FIGURE 2:**
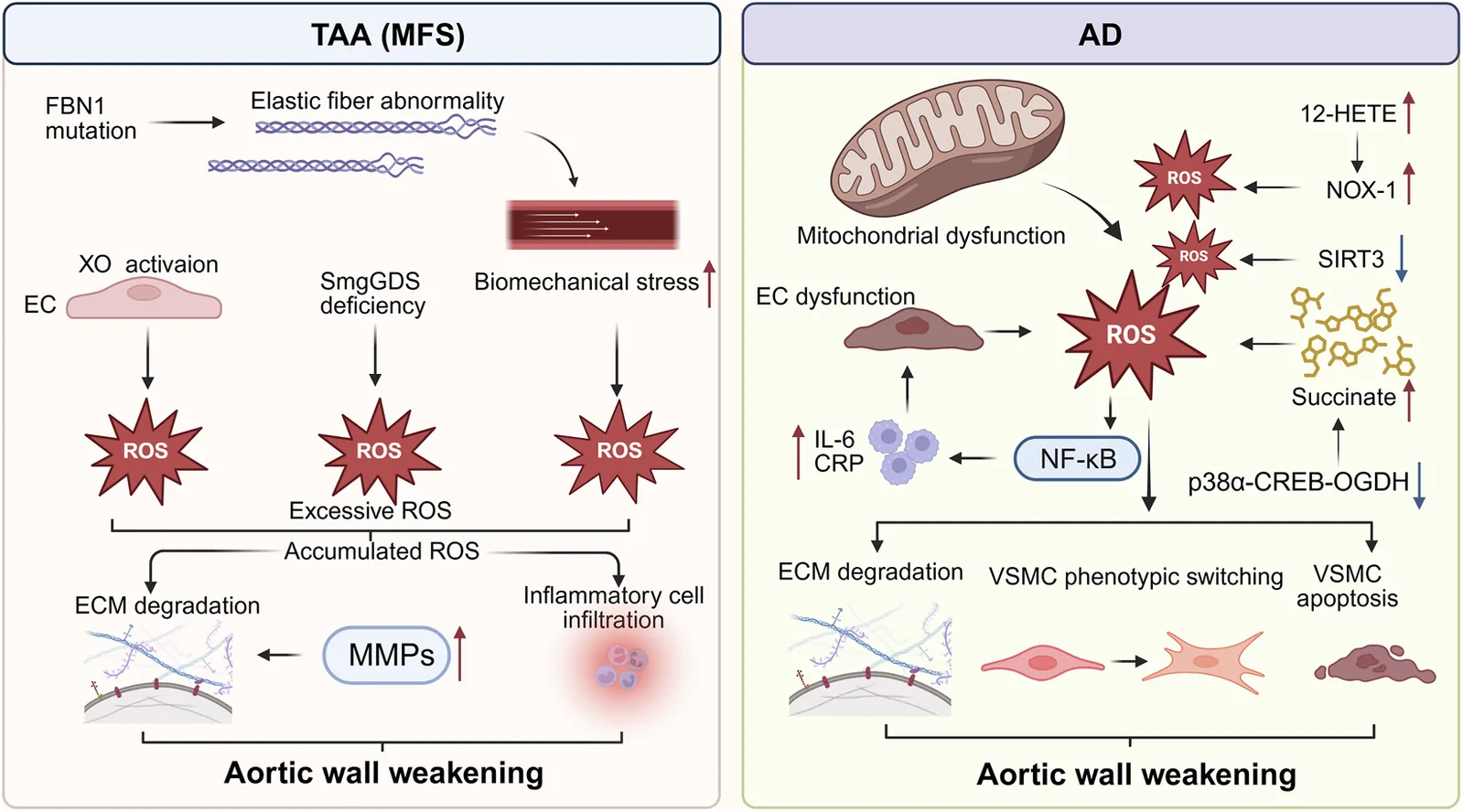
ROS-mediated oxidative stress mechanisms underlying aortic wall weakening in TAA and AD. In TAA, particularly MFS-associated TAA, FBN1 mutations cause elastic fiber abnormalities, increasing susceptibility to hemodynamic and biomechanical stress and promoting ROS overproduction. In ECs, XO activation further elevates ROS, while SmgGDS deficiency exacerbates ROS accumulation, MMP activation, and inflammatory cell infiltration. Excess ROS drives ECM degradation and inflammatory remodeling, ultimately weakening the aortic wall. In AD, mitochondrial dysfunction is a major source of ROS accumulation. Elevated ROS activates NF-κB signaling and increases inflammatory mediators such as IL-6 and CRP, aggravating EC dysfunction and forming a positive feedback loop of oxidative stress and inflammation. In parallel, increased 12-HETE activates the NOX-1/ROS/NF-κB axis, promoting cytokine release and immune cell recruitment. Downregulation of SIRT3 and dysregulation of the p38α-CREB-OGDH pathway further enhance mitochondrial oxidative stress, partly via succinate accumulation and increased succinate oxidation. Collectively, these events promote ECM degradation, VSMC phenotypic switching, and VSMC apoptosis, driving aortic wall destruction and AD progression. Created in https://BioRender.com. AD, aortic dissection; CRP, C-reactive protein; ECM, extracellular matrix; ECs, endothelial cells; FBN1, fibrillin 1; IL-6, interleukin-6; MFS, Marfan syndrome; MMPs, matrix metalloproteinases; NF-κB, nuclear factor-ĸB; NOX-1, NADPH oxidase 1; SIRT3, sirtuin 3; SmgGDS, Rap1 GTPase-GDP dissociation stimulator 1; TAA, thoracic aortic aneurysm; XO, xanthine oxidase and 12-HETE, 12-hydroxyeicosatetraenoic acid.

While oxidative stress is a shared pathological hallmark of both TAA and AD, the precise origins of ROS and their downstream effector pathways exhibit distinct profiles in each condition (Figure 2).

In TAA, oxidative stress has been particularly associated with heritable aortopathies such as MFS (Jiménez-Altayó et al., 2018). FBN1 mutations disrupt elastic fiber formation and alter the mechanical properties of the aortic wall, which may increase susceptibility to hemodynamic stress and disturb vascular redox homeostasis. In MFS mouse models, vascular dysfunction is accompanied by increased expression of oxidative enzymes, including xanthine oxidase (XO), and reduced antioxidant capacity, indicating a shift toward a pro-oxidative environment (Rodríguez-Rovira et al., 2022). XO-mediated oxidation of hypoxanthine and xanthine generates superoxide and hydrogen peroxide, contributing to vascular oxidative stress. Evidence from patients with MFS further supports the presence of redox imbalance in human disease, as reduced plasma total antioxidant capacity has been reported in these patients (Pérez-Torres et al., 2022). In addition to altered XO-related redox regulation, the Rap1 GTPase-GDP dissociation stimulator 1 (SmgGDS) has been implicated in maintaining vascular redox homeostasis. In Ang II-induced mouse model of AD, SmgGDS deficiency promotes aortic dilation, oxidative stress, elastic fiber disruption, increased matrix metalloproteinase (MMP) activity, and inflammatory cell infiltration, whereas restoration of SmgGDS expression attenuates these pathological changes (Nogi et al., 2018).

In AD, oxidative stress appears to be closely associated with mitochondrial ROS production and inflammatory signaling within the aortic wall. Excessive mitochondrial ROS can activate the nuclear factor-κB (NF-κB) pathway and increase the expression of inflammatory mediators such as interleukin 6 and C-reactive protein, thereby contributing to vascular inflammation and remodeling during AD progression (Xu et al., 2024). Arachidonic acid metabolism provides an additional link between oxidative stress and inflammation in AD. Aortic tissues from patients with AD exhibit increased levels of 12-hydroxyeicosatetraenoic acid (12-HETE) and upregulation of 12/15-lipoxygenase (12/15-LOX). Mechanistic studies indicate that macrophage-derived 12-HETE activates BLT2 signaling and the NOX-1/ROS/NF-κB cascade, promoting local inflammation, immune cell infiltration, and VSMC phenotypic switching (Li et al., 2026).

Mitochondrial redox regulation in AD is also associated with SIRT3, a mitochondrial NAD^+^-dependent deacetylase involved in metabolic regulation and electron transport chain (ETC) function (Xian et al., 2025). In Ang II-induced mouse models of AD, aortic SIRT3 expression is reduced, and SIRT3 deficiency leads to greater aortic dilation and a higher rate of aortic rupture, accompanied by increased aortic ROS accumulation and enhanced VSMC apoptosis (Qiu et al., 2021). In addition, altered succinate metabolism represents another potential source of mitochondrial oxidative stress in AD. Dysregulation of the p38α-CREB-OGDH pathway has been linked to changes in succinate metabolism. Elevated plasma succinate levels have been detected in patients with AD (Cui et al., 2021; Xu et al., 2023). Further *in vitro* studies suggest that extracellular succinate can be taken up by macrophages and VSMCs, where its oxidation through succinate dehydrogenase (SDH) contributes to mitochondrial ROS production and may influence vascular remodeling (Cui et al., 2021). Thus, the identification of altered succinate metabolism in patients with AD, combined with mechanistic studies demonstrating its contribution to mitochondrial ROS production and vascular remodeling, provides a rationale for targeting the succinate-SDH pathway as a potential strategy to restore mitochondrial redox balance and limit AD progression.

### Mitochondrial dynamics imbalance and AAD

**FIGURE 3:**
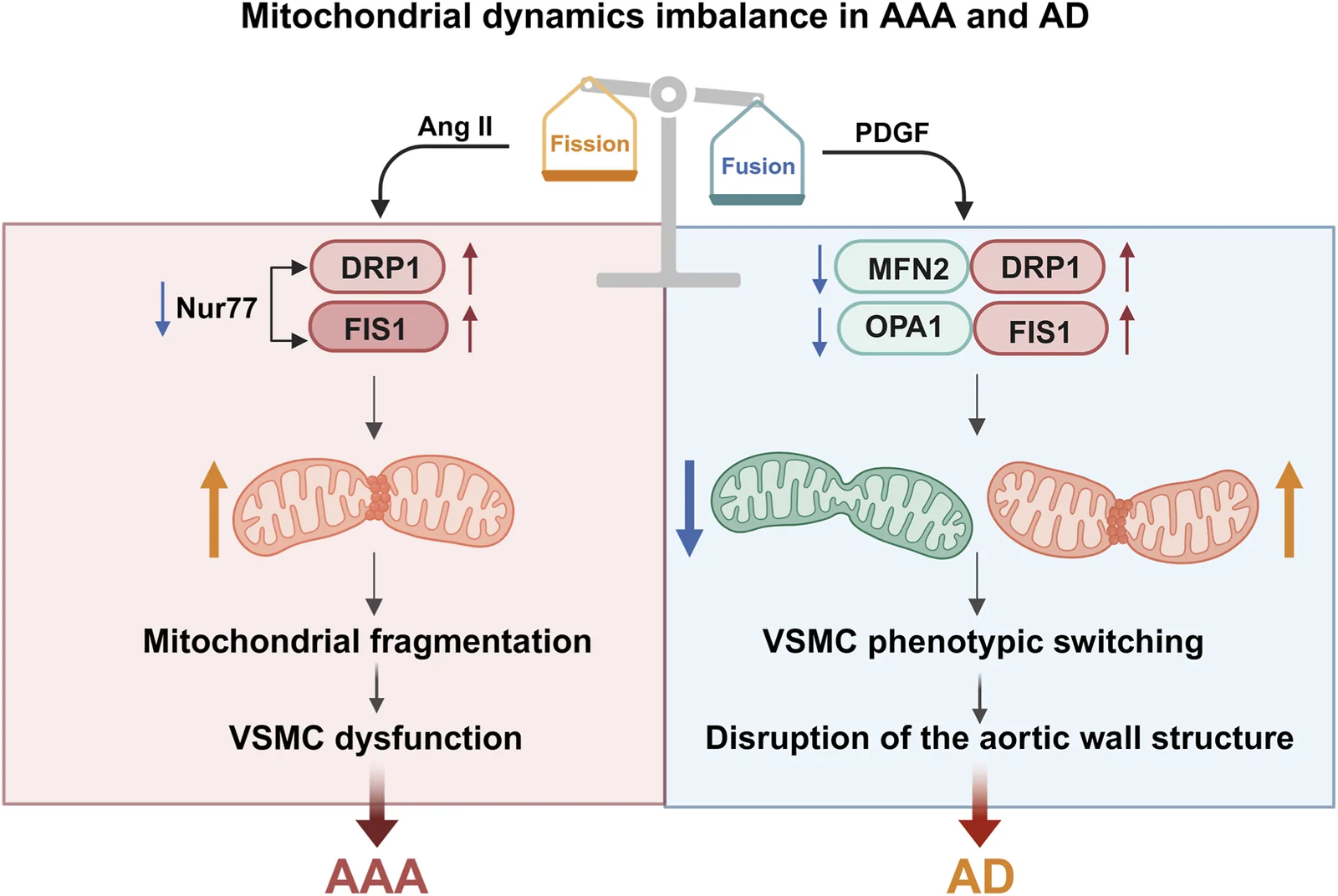
Imbalanced mitochondrial dynamics contributes to AAD progression by promoting VSMC dysfunction and aortic wall disruption. Mitochondrial fission and fusion are normally balanced to preserve mitochondrial homeostasis. In AAA, Ang II stimulation shifts mitochondrial dynamics toward excessive fission through DRP1 activation. Reduced Nur77 expression weakens the suppression of DRP1 and FIS1, resulting in increased mitochondrial fragmentation. This alteration is associated with VSMC dysfunction and acquisition of a senescence-associated inflammatory phenotype. Similar mitochondrial changes have also been observed in AD. PDGF signaling decreases the expression of fusion-related proteins, including MFN2 and OPA1, while increasing DRP1 and FIS1 levels, leading to mitochondrial fragmentation and VSMC switch toward a synthetic phenotype. These alterations may disturb aortic wall homeostasis and contribute to AD progression. Created in https://BioRender.com. AAA, abdominal aortic aneurysm; Ang II, angiotensin II; DRP1, dynamin-related protein 1; FIS1, fission protein 1; MFN2, mitofusin 2; OPA1, optic atrophy protein 1 and PDGF, platelet-derived growth factor.

Mitochondrial fission and fusion are essential for maintaining mitochondrial morphology and integrity. Disruption of this balance can impair mitochondrial quality control and alter mitochondrial function in vascular cells (Quintana-Cabrera and Scorrano, 2023). Altered mitochondrial dynamics have been observed in both AAA and AD (Figure 3).

In AAA, altered mitochondrial dynamics are predominantly characterized by enhanced mitochondrial fission. Increased DRP1 expression and mitochondrial fragmentation have been observed in an Ang II/β-aminopropionitrile (BAPN)-induced mouse model of AAA and in human AAA tissues, supporting a role for excessive mitochondrial fission in AAA pathology (Gutierrez et al., 2020; Cooper et al., 2021). Mechanistic studies in VSMCs indicate that Ang II promotes DRP1-dependent mitochondrial fragmentation and ROS accumulation, changes associated with inflammatory activation and VSMC phenotypic remodeling (Cooper et al., 2021). Consistent with these findings, pharmacological inhibition or genetic deletion of DRP1 attenuates aortic injury and reduces AAA formation in mice (Cooper et al., 2021). In murine models of AAA, treatment with the mitochondrial fission inhibitor Mdivi-1 attenuates mitochondrial fragmentation, inflammatory cell infiltration, and AAA development (Cooper et al., 2021). The nuclear receptor subfamily 4 group A member 1 (Nur77) provides an additional regulatory link between mitochondrial dynamics and VSMC dysfunction in AAA (Crean and Murphy, 2021). In experimental AAA models, reduced Nur77 expression has been observed in VSMCs (Geng et al., 2022). Nur77 deficiency increases the expression of the fission-related proteins FIS1 and DRP1, whereas the levels of the fusion-related proteins OPA1 and MFN2 remain relatively unchanged. This imbalance is accompanied by increased mitochondrial fragmentation and impaired mitochondrial network integrity, suggesting that loss of Nur77 may favor excessive mitochondrial fission during AAA development (Geng et al., 2022).

In AD, mitochondrial dynamics are also perturbed, with current evidence indicating a concomitant dysregulation of both mitochondrial fusion and fission. Mechanistic studies in cultured VSMCs have linked reduced mitochondrial fusion to phenotypic switching, including loss of contractile characteristics and acquisition of a synthetic phenotype (Zeng et al., 2024). Platelet-derived growth factor stimulation reduces MFN2 expression by approximately 50% and promotes mitochondrial fragmentation in VSMCs (Zeng et al., 2024). Evidence from human AD aortic tissues further supports the presence of disturbed mitochondrial dynamics in human disease. In the medial layer of affected aortas, reduced cellularity and disrupted ECM organization are accompanied by decreased expression of the fusion-related proteins MFN2 and OPA1 and increased expression of the fission-related proteins DRP1 and FIS1 (Wang et al., 2024). Clinical observations and experimental studies establish mitochondrial dynamics as an important regulator of VSMC behavior in AD, providing a mechanistic basis for targeting mitochondrial network remodeling as a potential therapeutic approach.

## Potential therapeutic targets

Surgeons can repair AAD, but pharmacotherapy remains a blank slate (Liu et al., 2026). We simply lack drugs capable of halting or reversing aortic remodeling. This clinical void has forced a deeper look into the molecular machinery driving vascular wall decay. Now, a steady stream of data points directly to mitochondrial dysfunction as a prime culprit in both structural remodeling and cellular failure. Consequently, targeting mitochondrial pathways is no longer just an academic exercise; it has become a central focus for identifying novel therapeutic entry points. To better bridge the mechanistic findings with clinical applications, the major mitochondrial targets, associated interventions, and their current translational status are summarized in Supplementary Table S1.

### Targeting mitochondrial energy metabolism and biogenesis

**FIGURE 4:**
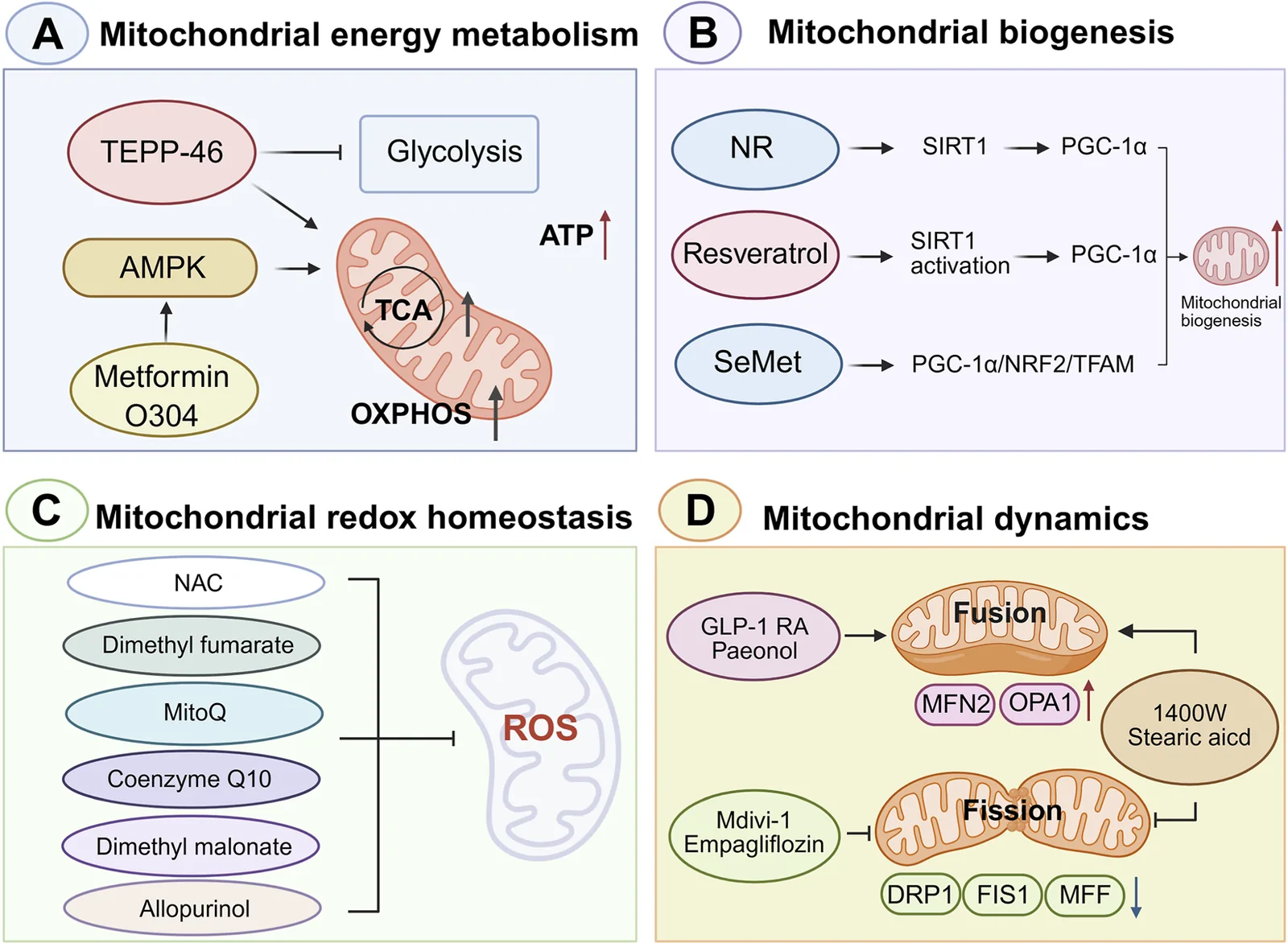
Mitochondria-targeted interventions in AAD. **(A)** Regulation of mitochondrial energy metabolism. Metformin and O304 activate AMPK signaling, promoting fatty acid oxidation and mitochondrial metabolic activity, with possible effects on ATP production and VSMC phenotypic regulation. TEPP-46 promotes PKM2 tetramerization and helps normalize the glycolysis-OXPHOS balance during vascular remodeling. **(B)** Promotion of mitochondrial biogenesis. NR enhances SIRT1/3 signaling and increases PGC-1α expression. SeMet activates the PGC-1α/NRF2/TFAM axis and promotes mitochondrial biogenesis. Resveratrol suppresses Ang II-associated signaling and has also been reported to enhance mitochondrial biogenic activity, potentially contributing to vascular protection. **(C)** MitoQ, coenzyme Q10, allopurinol and dimethyl malonate decrease ROS levels and modulate oxidative stress responses. NAC and dimethyl fumarate enhance endogenous antioxidant mechanisms and may alleviate oxidative stress-related vascular injury in AAD. **(D)** Regulation of mitochondrial dynamics. Mdivi-1 and empagliflozin reduce excessive mitochondrial fission through DRP1-associated mechanisms. GLP-1 RA and paeonol modulate mitochondrial fusion by affecting MFN2 and OPA1. Other compounds, including 1400W and stearic acid, may influence mitochondrial dynamics by shifting the balance between fission and fusion. Created in https://BioRender.com. AMPK, AMP-activated protein kinase; GLP-1 RA, GLP-1 receptor agonists; MitoQ, Mitoquinone; MFF, mitochondrial fission factor; NAC, N-acetylcysteine; NR, nicotinamide riboside; PKM2, pyruvate kinase M2; SeMet, selenomethionine; SIRT 1, sirtuin1 and TCA, tricarboxylic acid.

Metabolic dysregulation has emerged as a potential therapeutic target in AAA and AD (Figure 4A).

In AAA, metformin is among the most extensively studied metabolism-modulating agents. Metformin activates AMPK signaling, partly through inhibition of mitochondrial complex I, although AMPK-independent effects have also been reported. AMPK activation suppresses acetyl-CoA carboxylase activity and promotes fatty acid oxidation, while inhibition of mTORC1 limits anabolic signaling under metabolic stress (Hatmal et al., 2025). In a rat model of AAA, metformin activates AMPK signaling and suppresses mTOR pathway activity, accompanied by reduced macrophage infiltration, MMP expression, neovascularization, and preservation of the contractile VSMC phenotype (He et al., 2021). Thus, its protective effects in AAA may involve both mitochondrial/metabolic regulation and broader anti-inflammatory actions. Importantly, metformin is a widely prescribed agent for type 2 diabetes, and observational studies have associated metformin use with slower AAA growth (Yu et al., 2019). Other AMPK-modulating compounds have also been examined in experimental aortic diseases. O304, a small-molecule AMPK activator, increases AMPKα phosphorylation at Thr172 and enhances the expression of mitochondrial biogenesis-related proteins, including PGC-1α and TFAM (Zhu et al., 2022). In an Ang II-infused mouse model of AAA, O304 reduces aortic dilation and blood pressure elevation, together with decreased MMP2, MMP3, and MMP9 expression and changes consistent with reduced VSMC phenotypic switching (Sun and Du, 2024). These findings support AMPK activation as a promising strategy for targeting metabolic dysfunction in AAA.

In AD, metabolic reprogramming of VSMCs is closely involved in disease progression. Membrane-associated RING-CH-type finger 2 (March2) expression is reduced in VSMCs from AD patients and experimental models. March2 promotes K33-linked ubiquitination of pyruvate kinase M2 (PKM2), favoring its tetrameric form and helping maintain oxidative metabolism. Loss of March2 shifts PKM2 toward the dimeric form, with increased glycolysis and lactate production, H3K18 acetylation, and p53-dependent apoptotic signaling (Zahra et al., 2020). Targeting this metabolic shift with TEPP-46, a PKM2 activator that stabilizes tetramer formation, reduces excessive glycolysis, lactate accumulation, histone hyperacetylation, and VSMC apoptosis (Li Y. E. et al., 2025). In BAPN-induced mouse models of AD, TEPP-46 attenuates vascular remodeling and medial elastic fiber disruption in March2-deficient AD models and partially restores the VSMC phenotype (Li Y. E. et al., 2025). While current experimental studies provide encouraging support for TEPP-46 in AD, its clinical efficacy and safety profiles have yet to be established. Therefore, translating these preclinical findings into human cohorts represents an important avenue for future clinical research.

### Promoting mitochondrial biogenesis

Impaired mitochondrial biogenesis has been implicated in vascular cell dysfunction during AAA, TAA, and AD progression, and several interventions targeting NAD^+^ metabolism and PGC-1α-related regulatory networks have been explored to restore mitochondrial homeostasis (Figure 4B).

In hereditary TAA, particularly MFS-associated aortic disease, restoration of mitochondrial biogenesis has emerged as a potential therapeutic strategy. Nicotinamide riboside (NR), an NAD^+^ precursor, enhances intracellular NAD^+^ availability and promotes SIRT1-dependent activation of PGC-1α, thereby stimulating mitochondrial biogenesis (Qu et al., 2023). In *Fbn1*-deficient VSMCs, NR treatment improves mitochondrial respiratory capacity and increases mtDNA content, accompanied by restoration of contractile markers, including *MYH11* and *CNN1*, and attenuation of aortic dilation (Oller et al., 2021). Similarly, resveratrol, a SIRT1 activator with antioxidant properties, has been investigated as another approach to enhance SIRT1-related mitochondrial regulation. In an exploratory single-arm clinical trial involving patients with MFS, resveratrol treatment was associated with a reduced rate of aortic root enlargement, providing preliminary clinical evidence for its potential benefit in hereditary TAA (van Andel et al., 2024). Together, these findings support NAD^+^-boosting strategies, including NR and resveratrol, as potential therapeutic approaches for hereditary TAA through restoration of mitochondrial homeostasis and protection of VSMC function.

Beyond hereditary TAA, mitochondrial biogenesis-related interventions have also been examined in experimental AAA models. In an Ang II-induced *ApoE* ^−/−^ mouse model of atherosclerotic AAA, impaired mitochondrial respiration and reduced mitochondrial protein expression were observed in VSMCs. Enhancing NAD^+^ availability with NR restored mitochondrial function and reduced aneurysm formation and aortic rupture (Oller et al., 2022).

In AD, the PGC-1α-related regulatory network has also been explored as a potential therapeutic target. Selenomethionine (SeMet) modulates the PGC-1α/NRF2/TFAM axis, accompanied by increased GPX4 expression, reduced lipid peroxidation, and improved mitochondrial biogenesis. These effects are associated with suppression of ferroptosis-related VSMC phenotypic switching. In BAPN-induced mouse models of AD, SeMet treatment reduces aortic dilation, elastic fiber disruption, collagen accumulation, and MMP2/MMP9 expression (Lai et al., 2026). These findings suggest that SeMet may influence aortic remodeling through coordinated regulation of mitochondrial maintenance and ferroptosis.

### Maintaining mitochondrial redox homeostasis

Oxidative imbalance is frequently observed during vascular remodeling, and several interventions targeting mitochondrial ROS or related redox pathways have been investigated as potential therapeutic strategies in AAD (Figure 4C).

Mitoquinone (MitoQ), a mitochondria-targeted ubiquinone derivative, represents a potential strategy to counteract mitochondrial oxidative stress by selectively accumulating within mitochondria and scavenging mitochondrial ROS. Clinical studies have provided preliminary evidence supporting the cardiovascular benefits of MitoQ. For example, MitoQ supplementation improved endothelial function by reducing mitochondria-derived oxidative stress in individuals with cardiovascular risk factors (Rossman et al., 2018). Beyond direct ROS scavenging, modulation of mitochondrial metabolic pathways represents another approach to limit oxidative stress in AD. Increased circulating succinate levels have been detected in patients with AD, while succinate accumulation within the aortic wall may promote mitochondrial ROS production through SDH. In a BAPN- and Ang II-induced mouse model of AD, inhibition of succinate oxidation with dimethyl malonate reduces mitochondrial ROS accumulation and attenuates vascular injury, highlighting the succinate-SDH axis as a potential metabolic target for redox regulation in AD (Cui et al., 2021).

In addition to mitochondria-targeted antioxidants, interventions aimed at restoring mitochondrial respiratory function have also shown therapeutic potential. Coenzyme Q10 (CoQ10), an essential electron carrier within the mitochondrial, ETC, improves mitochondrial bioenergetics and redox balance. In a BAPN-induced mouse model of AD, CoQ10 treatment rapidly restored mitochondrial function, promoted re-establishment of the contractile VSMC phenotype, improved aortic function, and enhanced survival (Luo et al., 2024). *In vitro*, CoQ10 treatment improves mitochondrial function and reduces oxidative stress (Zhang et al., 2024). Similarly, N-acetylcysteine (NAC) enhances endogenous antioxidant capacity by increasing glutathione availability, providing another strategy to restore redox balance under vascular stress.

Beyond mitochondrial ROS, ROS-generating enzymes represent additional disease-specific targets. In hereditary TAA associated with MFS, increased xanthine oxidoreductase (XOR) expression and XO activity contribute to vascular oxidative stress. Pharmacological inhibition of XOR with allopurinol attenuated aortic root aneurysm progression in *Fbn1* ^C1041G/+^ mice by reducing H_2_O_2_ production, endothelial dysfunction, elastic fiber fragmentation, and MMP2 overexpression (Rodríguez-Rovira et al., 2022).

Endogenous antioxidant signaling may provide another therapeutic route. The KEAP1-NRF2-ARE pathway represents a major endogenous defense mechanism against oxidative stress and regulates the expression of antioxidant and cytoprotective genes (Cuadrado et al., 2018). Dimethyl fumarate, an NRF2 activator approved for the treatment of multiple sclerosis, demonstrates the clinical feasibility of pharmacological NRF2 activation (Gold et al., 2012).

Overall, redox-targeted interventions represent a promising therapeutic direction for AAD by regulating mitochondrial ROS generation, restoring antioxidant capacity, and modulating metabolic pathways involved in oxidative stress responses.

### Regulating mitochondrial dynamics

Restoring mitochondrial network stability has been explored as a potential strategy for limiting mitochondrial dysfunction during AAA, TAA and AD progression (Figure 4D).

In AAA, excessive mitochondrial fission, particularly DRP1-dependent fragmentation, represents a potential therapeutic target. In an Ang II- and BAPN-induced mouse model of AAA, pharmacological inhibition of mitochondrial fission with Mdivi-1 reduces mitochondrial fragmentation and attenuates aneurysm formation (Cooper et al., 2021). In cultured VSMCs, Mdivi-1 also attenuates Ang II-induced phenotypic switching (Cooper et al., 2021). Beyond DRP1 inhibition, Mdivi-1 may also influence additional cellular pathways, suggesting that its vascular protective effects may involve broader regulation of mitochondrial network stability (Bordt et al., 2017). Empagliflozin, a sodium-glucose cotransporter 2 inhibitor, has been reported to regulate DRP1- and FIS1-associated mitochondrial fission and improve endothelial barrier function in experimental studies (Zou et al., 2022). In Ang II-infused male *ApoE* ^−/−^ mice, empagliflozin reduced mitochondrial fragmentation by modulating fission-related pathways, accompanied by decreased oxidative stress, inflammation, and VSMC apoptosis (Ortega et al., 2019).

Beyond direct modulation of mitochondrial fission machinery, metabolic receptor pathways have also been implicated in the regulation of mitochondrial dynamics. Similarly, GLP-1 receptor activation has been shown to improve mitochondrial morphology, membrane potential, and OXPHOS activity in cultured VSMCs, accompanied by regulation of MFN2 expression and PKA-dependent DRP1 signaling (Torres et al., 2016). Tirzepatide, a dual GIP/GLP-1 receptor agonist clinically used for metabolic disorders, has recently been investigated in experimental AAA. In an Ang II-infused *ApoE* ^−/−^ mouse model, tirzepatide attenuated AAA development by improving endothelial dysfunction, preserving elastin integrity, reducing neovascularization and macrophage accumulation, and suppressing inflammatory signaling pathways (Gómez-Martín et al., 2026).

In TAA, mitochondrial dynamics have also emerged as a potential therapeutic target, particularly in genetic aortopathies such as MFS. Increased iNOS expression and altered NO signaling contribute to aortic pathology in MFS mouse models (de la Fuente-Alonso et al., 2021). Treatment with the selective iNOS inhibitor 1400W modulates mitochondrial fusion abnormalities and partially restores the fission-fusion balance (Zheng et al., 2025).

In AD, stearic acid has been reported to regulate JNK/MAPK signaling and VSMC phenotype while reducing excessive mitochondrial fission and improving mitochondrial ultrastructure in a BAPN-induced mouse model of AD and Ang II-treated VSMCs (Wang et al., 2024).

Beyond AAD-specific interventions, additional compounds targeting mitochondrial fusion pathways have provided mechanistic support for this therapeutic concept. Paeonol has been reported to promote MFN2-mediated mitochondrial fusion through the PKCε-STAT3 pathway, accompanied by reduced oxidative stress and improved cardiac function in experimental doxorubicin-induced cardiotoxicity (Ding M. et al., 2023).

Overall, modulation of mitochondrial dynamics represents a promising therapeutic strategy for restoring mitochondrial network stability during AAD progression.

### Translational limitations of mitochondria-targeted interventions

While experimental models have provided compelling insights into the role of mitochondrial dysfunction in AAD, the translation of mitochondria-targeted therapies into clinical practice remains highly challenging. Several critical translational limitations must be addressed, particularly regarding target specificity, off-target effects, mitochondrial delivery, and the lack of robust clinical evidence.

A primary impediment in current mitochondrial pharmacology lies in achieving precise target specificity. Many compounds exhibit broad systemic effects rather than acting exclusively on mitochondria. For instance, the vascular benefits of metformin and O304 are likely mediated by broader metabolic and inflammatory changes rather than isolated AMPK activation (Triggle et al., 2022; Norlin et al., 2023). Similarly, resveratrol affects multiple mechanisms beyond mitochondrial biogenic pathways (Cheng et al., 2020). Furthermore, Mdivi-1, a mitochondrial fission inhibitor, exhibits antioxidative capacity in a relatively DRP1-independent manner, indicating that its effects are not restricted to modifying mitochondrial morphology (Duan et al., 2020).

Consequently, these broad mechanisms increase the risk of detrimental off-target effects and physiological disruption. Targeting DRP1 to broadly inhibit mitochondrial fission poses significant translational risks, given that physiological fission is necessary to detach damaged organelles from the healthy network for mitophagic degradation and overall quality control (Quiles and Gustafsson Å, 2022). Likewise, indiscriminately reducing ROS may not yield beneficial outcomes, as it can inadvertently suppress the essential physiological signaling functions of ROS (Sies and Jones, 2020).

Delivery efficacy poses another significant barrier. The application of promising antioxidant agents like NAC is heavily restricted by limitations in bioavailability and targeted mitochondrial delivery (Zhang Y. et al., 2026). To overcome these pharmacological barriers, nanocarrier-based strategies, including liposomal systems and other nanotechnology platforms, are currently being developed to improve tissue distribution and precise mitochondrial accumulation (Chen L. et al., 2025; Wang et al., 2025).

Finally, the field suffers from a profound lack of robust clinical evidence. Direct evidence supporting the use of CoQ10 or NAC in human AAD is still limited. While pharmacological regulation of antioxidant signaling is feasible, whether such approaches can actually modify human aortic degeneration remains to be determined. Although preclinical data firmly link defective mitochondrial biogenesis to AAD, it is still uncertain whether simply boosting this process will halt disease progression in patients, given the highly integrated and complex nature of vascular remodeling. Future research should therefore prioritize highly selective delivery strategies, clinically relevant models, and well-designed clinical trials to determine whether mitochondrial modulation can ultimately provide meaningful disease modification in patients with AAD.

## Conclusion

Currently, the clinical management of AAD remains predominantly reliant on surgical repair and endovascular interventions. Effective disease-modifying pharmacological therapies are still lacking (Liu et al., 2026). Despite the growing body of experimental evidence linking mitochondrial defects to aortic wall degeneration, the precise mechanisms by which these abnormalities drive specific pathological events remain poorly defined. Mitochondria regulate cellular metabolism and stress responses beyond ATP production (Harrington et al., 2023). In VSMCs, mitochondrial metabolic dysfunction and excessive oxidative stress have been implicated in phenotypic remodeling, inflammatory activation, and apoptotic loss during AAD progression (Luo et al., 2024). These alterations are associated with metabolic reprogramming, including altered TCA cycle activity and mitochondrial dysfunction, which may contribute to programmed cell death (Luo et al., 2024).

We must view mitochondrial dysfunction in AAD not as a single, isolated fault, but as a coordinated disturbance involving multiple regulatory processes. Dysregulated metabolism, disrupted biogenesis, redox imbalance, and fragmented dynamics all converge to compromise VSMC function and weaken aortic wall integrity. Yet, whether restoring mitochondrial function alone is sufficient to halt or reverse AAD progression remains uncertain. Vascular remodeling is a multifactorial process involving complex cellular and molecular alterations that extend beyond mitochondrial regulation.

Moving these mitochondrial targets from bench to bedside remains a formidable challenge. Successful translation will require a deeper understanding of cell-specific mitochondrial responses, particularly because ECs and VSMCs possess distinct metabolic characteristics and may respond differently to mitochondrial stress. Moreover, challenges including mitochondrial delivery efficiency, target specificity, potential off-target effects, and limited clinical evidence remain major barriers to therapeutic development. Importantly, whether mitochondrial abnormalities represent primary drivers of AAD or secondary consequences of vascular injury remains unresolved. Future studies integrating cell-specific approaches, improved mitochondrial targeting strategies, and clinically relevant models will be essential to define the therapeutic potential of mitochondria-targeted interventions.

## Glossary

AA: aortic aneurysm
AAA: abdominal aortic aneurysm
AAD: aortic aneurysm and dissection
ACO2: aconitase 2
AD: aortic dissection
ALOX12: arachidonate 12-lipoxygenase
AMPK: AMP-activated protein kinase
Ang II: angiotensin II
ATP: adenosine triphosphate
BAPN: β-aminopropionitrile
CoQ10: Coenzyme Q10
DRP1: dynamin related protein 1
ECM: extracellular matrix
ECs: endothelial cells
ETC: electron transport chain
FBN1: fibrillin 1
FIS1: mitochondrial fission 1 protein
H4K16la: histone H4 Lysine 16 lactylation
LDS: Loeys-Dietz syndrome
March2: membrane-associated RING-CH finger protein 2
MFF: mitochondrial fission factor
MFN1: mitofusin 1
MFN2: mitofusin 2
MFS: Marfan syndrome
MiD49/51: mitochondrial dynamics protein of 49/51
MitoQ: Mitoquinone
MMP: matrix metalloproteinases
mtDNA: mitochondrial DNA
NAC: N-acetylcysteine
NAD: nicotinamide adenine dinucleotide
NADH: reduced nicotinamide adenine dinucleotide
NCOR1: nuclear receptor corepressor 1
NFE2L2: NFE2 like bZIP transcription factor 2
NF-κB: nuclear factor-ĸB
NOX: NADPH oxidases
NR: nicotinamide riboside
NR1D1: nuclear receptor subfamily 1 group D member 1
NRF1: nuclear respiratory factor 1
NRF2: nuclear respiratory factor 2
Nur77: nuclear receptor subfamily 4 group A member 1
OMM: outer mitochondrial membrane
OPA1: optic atrophy protein 1
OXPHOS: oxidative phosphorylation
PAH: pulmonary arterial hypertension
PDK1: pyruvate dehydrogenase kinase 1
PDK4: pyruvate dehydrogenase kinase 4
PGC-1α: peroxisome proliferator-activated receptor gamma coactivator 1-alpha
PKM2: pyruvate kinase M2
ROS: reactive oxygen species
SeMet: selenomethionine
SDH: succinate dehydrogenase
SIRT1: sirtuin 1
SIRT3: sirtuin 3
SIRT6: sirtuin 6
SmgGDS: Rap1 GTPase-GDP dissociation stimulator 1
TAA: thoracic aortic aneurysm
TCA: tricarboxylic acid
TFAM: transcription factor A, mitochondrial
VSMC: vascular smooth muscle cell
XO: xanthine oxidase
XOR: xanthine oxidoreductase
12/15-LOX: 12/15-lipoxygenase
12-HETE: 12-hydroxyeicosatetraenoic acid
