# Supplementary material for: Mitochondrial dysfunction in aortic aneurysm and dissection: mechanisms and therapeutic implications
Source: Front Pharmacol. 2026 Sep 10;17:1942911. doi: 10.3389/fphar.2026.1942911 (PMC13600970; doi:10.3389/fphar.2026.1942911)
Supplement: Supplementary file 1 [file Table1.docx]

**Supplemental Material**

**Mitochondrial Dysfunction in Aortic Aneurysm and Dissection: Mechanisms and Therapeutic Implications**

**Authors**

Yang Yang^1,#^; Zhili He^1,#^; Yan Wang^1^; Shuang Zhao^2,^*; Tianyu Song^1,^*.

**Affiliations**

^1^School of Medicine, Nanjing University of Chinese Medicine, Nanjing, China;

^2^Key Laboratory of Drug Targets and Translational Medicine for Cardio-cerebrovascular Diseases; Medical Basic Research Innovation Center for Cardiovascular and Cerebrovascular Diseases, Ministry of Education; School of Pharmacy, Nanjing Medical University, Nanjing, China.

^#^These authors contributed equally

**Supplemental Table S1. Major mitochondrial targets, relevant interventions, experimental models, and translational status in AAD.**

| **Major Mitochondrial Targets** | **Relevant Interventions** | **Experimental Models** | **Level of Evidence** | **Translational Status** | **Refs.** |
| --- | --- | --- | --- | --- | --- |
| Mitochondrial energy metabolism | Metformin | • In vivo: PPE-induced male Sprague-Dawley rats (AAA)  • Human: observational studies in patients with AAA | Preclinical; observational clinical study | FDA-approved for T2DM; ongoing clinical trials for AAA progression | (Yu et al., 2019; He et al., 2021) |
|  | O304 | • In vivo: Ang II-infused male *ApoE*^-/-^ mice (AAA) | Preclinical | Preclinical for AAA | (Sun and Du, 2024) |
|  | TEPP-46 | • In vivo: BAPN-induced mice (AD) | Preclinical | Preclinical for AD | (Le et al., 2020; Li et al., 2025) |
| Mitochondrial biogenesis | NR | • In vitro: in Fbn1-deficient VSMCs (TAA)  • In vivo: Ang II-infused male *ApoE*^-/-^ mice fed a Western diet (AAA) | Preclinical | Preclinical for AAA and TAA | (Oller et al., 2021; Oller et al., 2022) |
|  | Resveratrol | • In vitro: Ang II-treated cultured VSMCs  • In vivo: Ang II-infused *ApoE*^-/-^ mice (AAA) • Human：MFS patients | Preclinical; observational clinical study | Preliminary human evidence | (Moran et al., 2017; van Andel et al., 2024; Qu et al., 2025) |
|  | SeMet | • In vivo: BAPN-induced mice (AD) | Preclinical | Preclinical for AD | (Li et al., 2019; Wu et al., 2024) |
| Mitochondrial redox homeostasis | MitoQ | • Human: evaluating endothelial function and mitochondrial oxidative stress | Human cardiovascular studies | Promising mitochondria-targeted antioxidant strategy | (Rossman et al., 2018) |
|  | Dimethyl malonate | • In vivo: Ischemia-reperfusion and inflammatory models | Preclinical mechanistic evidence | Potential metabolic target for AD | (Cui et al., 2021) |
|  | CoQ10 | • In vitro: *FBN1*-knockdown human smooth muscle cells (MFS-associated TAA) • In vivo: BAPN-induced mice (AD) | Preclinical | Preclinical for TAA and AD | (Luo et al., 2024; Zhang et al., 2024) |
|  | Allopurinol | • In vivo: *Fbn1*^C1039G/+^ mice (TAA) | Preclinical | Preclinical for TAA | (Rodríguez-Rovira et al., 2022) |
|  | Dimethyl fumarate | • In vivo: PPE-induced male mice (AAA) • In vivo: BAPN-induced male mice (AD) | Preclinical | FDA-approved for multiple sclerosis; preclinical for AAA and AD | (Wang et al., 2025) |
| Mitochondrial dynamics | Mdivi-1 | • In vivo: Ang II- and BAPN-induced mice (AAA)  • In vitro: Ang II-treated cultured VSMCs | Preclinical | Preclinical for AAA | (Cooper et al., 2021) |
|  | 1400W | • In vivo: *Adamts1*^+/−^ and *Fbn1*^C1039G/+^ mice (TAA) | Preclinical | Preclinical for TAA | (Oller et al., 2017) |
|  | Stearic acid | • In vivo: BAPN-induced mice (AD)  • In vitro: Ang II-treated VSMCs | Preclinical | Preclinical for AD | (Wang et al., 2024) |
|  | Empagliflozin | • In vivo: Ang II-infused male *ApoE*^-/-^ mice (AAA) | Preclinical | FDA-approved for T2DM; preclinical for AAA | (Ortega et al., 2019) |
|  | GLP-1 RA | • In vivo: Ang II-infused male *ApoE*^-/-^ mice (AAA) • Human: Patients with TAA | Preclinical; observational clinical study | Ongoing clinical trials for TAA progression | (Gómez-Martín et al., 2026; Raslan et al., 2026) |
|  | Paeonol | • In vivo: PPE-induced male mice (AAA) | Preclinical | Preclinical for AAA | (Chen et al., 2021) |

**Abbreviations:** AAA, abdominal aortic aneurysm; AAD, aortic aneurysm and dissection; AD, aortic dissection; Ang II, angiotensin II; ApoE, apolipoprotein E; BAPN, β-aminopropionitrile; CoQ10, coenzyme Q10; FBN1, fibrillin-1; FDA, U.S. Food and Drug Administration; GLP-1 RA, glucagon-like peptide-1 receptor agonist; Mdivi-1, mitochondrial division inhibitor 1; MitoQ, Mitoquinone; MFS, Marfan syndrome; NR, nicotinamide riboside; PPE, porcine pancreatic elastase; SeMet, selenomethionine; TAA, thoracic aortic aneurysm; T2DM, type 2 diabetes mellitus; VSMC, vascular smooth muscle cell.

**References**

Chen, S., Luo, K., Bian, S., Chen, J., Qiu, R., Wu, X., et al. (2021). Paeonol Ameliorates Abdominal Aortic Aneurysm Progression by the NF-κB Pathway. *Ann. Vasc. Surg.* 77, 255-262. doi:10.1016/j.avsg.2021.06.003

Cooper, H. A., Cicalese, S., Preston, K. J., Kawai, T., Okuno, K., Choi, E. T., et al. (2021). Targeting mitochondrial fission as a potential therapeutic for abdominal aortic aneurysm. *Cardiovasc. Res.* 117, 971-982. doi:10.1093/cvr/cvaa133

Cui, H., Chen, Y., Li, K., Zhan, R., Zhao, M., Xu, Y., et al. (2021). Untargeted metabolomics identifies succinate as a biomarker and therapeutic target in aortic aneurysm and dissection. *Eur. Heart J.* 42, 4373-4385. doi:10.1093/eurheartj/ehab605

Gómez-Martín, Á., Marques, P., Arce-Recatalá, C., Vea, Á., Domingo, E., Alabadi, B., et al. (2026). Tirzepatide, a dual GIP/GLP-1 receptor agonist, attenuates endothelial dysfunction and angiotensin II-induced abdominal aortic aneurysm in ApoE(-/-) mice. *Pharmacol. Res.* 230, 108321. doi:10.1016/j.phrs.2026.108321

He, J., Li, N., Fan, Y., Zhao, X., Liu, C., and Hu, X. (2021). Metformin Inhibits Abdominal Aortic Aneurysm Formation through the Activation of the AMPK/mTOR Signaling Pathway. *J. Vasc. Res.* 58, 148-158. doi:10.1159/000513465

Le, S., Zhang, H., Huang, X., Chen, S., Wu, J., Chen, S., et al. (2020). PKM2 Activator TEPP-46 Attenuates Thoracic Aortic Aneurysm and Dissection by Inhibiting NLRP3 Inflammasome-Mediated IL-1β Secretion. *J. Cardiovasc. Pharmacol. Ther.* 25, 364-376. doi:10.1177/1074248420919966

Li, H., Xia, N., Hasselwander, S., and Daiber, A. (2019). Resveratrol and Vascular Function. *Int. J. Mol. Sci.* 20, doi:10.3390/ijms20092155

Li, Y. E., Liu, S., Wang, L., Du, Y., Wu, L., Chen, H., et al. (2025). March2 Alleviates Aortic Aneurysm/Dissection by Regulating PKM2 Polymerization. *Circ. Res.* 136, e73-e93. doi:10.1161/circresaha.124.325049

Luo, Y., Luo, J., An, P., Zhao, Y., Zhao, W., Fang, Z., et al. (2024). The activator protein-1 complex governs a vascular degenerative transcriptional programme in smooth muscle cells to trigger aortic dissection and rupture. *Eur. Heart J.* 45, 287-305. doi:10.1093/eurheartj/ehad534

Moran, C. S., Biros, E., Krishna, S. M., Wang, Y., Tikellis, C., Morton, S. K., et al. (2017). Resveratrol Inhibits Growth of Experimental Abdominal Aortic Aneurysm Associated With Upregulation of Angiotensin-Converting Enzyme 2. *Arterioscler. Thromb. Vasc. Biol.* 37, 2195-2203. doi:10.1161/atvbaha.117.310129

Oller, J., Gabandé-Rodríguez, E., Roldan-Montero, R., Ruiz-Rodríguez, M. J., Redondo, J. M., Martín-Ventura, J. L., et al. (2022). Rewiring Vascular Metabolism Prevents Sudden Death due to Aortic Ruptures-Brief Report. *Arterioscler. Thromb. Vasc. Biol.* 42, 462-469. doi:10.1161/atvbaha.121.317346

Oller, J., Gabandé-Rodríguez, E., Ruiz-Rodríguez, M. J., Desdín-Micó, G., Aranda, J. F., Rodrigues-Diez, R., et al. (2021). Extracellular Tuning of Mitochondrial Respiration Leads to Aortic Aneurysm. *Circulation* 143, 2091-2109. doi:10.1161/circulationaha.120.051171

Oller, J., Méndez-Barbero, N., Ruiz, E. J., Villahoz, S., Renard, M., Canelas, L. I., et al. (2017). Nitric oxide mediates aortic disease in mice deficient in the metalloprotease Adamts1 and in a mouse model of Marfan syndrome. *Nat. Med.* 23, 200-212. doi:10.1038/nm.4266

Ortega, R., Collado, A., Selles, F., Gonzalez-Navarro, H., Sanz, M. J., Real, J. T., et al. (2019). SGLT-2 (Sodium-Glucose Cotransporter 2) Inhibition Reduces Ang II (Angiotensin II)-Induced Dissecting Abdominal Aortic Aneurysm in ApoE (Apolipoprotein E) Knockout Mice. *Arterioscler. Thromb. Vasc. Biol.* 39, 1614-1628. doi:10.1161/atvbaha.119.312659

Qu, Y., Zhang, N., and Zhao, Y. (2025). Resveratrol Inhibits Abdominal Aortic Aneurysm Progression by Reducing Extracellular Matrix Degradation, Apoptosis, Autophagy, and Inflammation of Vascular Smooth Muscle Cells via Upregulation of HMOX1. *J. Endovasc. Ther.* 32, 1224-1236. doi:10.1177/15266028231202727

Raslan, M. A., Abdul Nabi, H., Dreher, L., Al Osta, S., De Sousa Barzon Serra, V., Ayoub, C., et al. (2026). The Association Between Glucagon-like Peptide-1 Receptor Agonists and Clinical Outcomes in Patients with Thoracic Aortic Aneurysm. *Diagnostics (Basel)* 16, doi:10.3390/diagnostics16111742

Rodríguez-Rovira, I., Arce, C., De Rycke, K., Pérez, B., Carretero, A., Arbonés, M., et al. (2022). Allopurinol blocks aortic aneurysm in a mouse model of Marfan syndrome via reducing aortic oxidative stress. *Free Radic. Biol. Med.* 193, 538-550. doi:10.1016/j.freeradbiomed.2022.11.001

Rossman, M. J., Santos-Parker, J. R., Steward, C. a. C., Bispham, N. Z., Cuevas, L. M., Rosenberg, H. L., et al. (2018). Chronic Supplementation With a Mitochondrial Antioxidant (MitoQ) Improves Vascular Function in Healthy Older Adults. *Hypertension* 71, 1056-1063. doi:10.1161/hypertensionaha.117.10787

Sun, D., and Du, Y. (2024). O304 alleviates abdominal aortic aneurysm formation via AMPK/mTOR/MMP pathway activation. *Front. Pharmacol.* 15, 1457817. doi:10.3389/fphar.2024.1457817

Van Andel, M. M., Bosshardt, D., Schrauben, E. M., Merton, R., Van Kimmenade, R. R. L., Scholte, A., et al. (2024). Effects of resveratrol on aortic growth in patients with Marfan syndrome: a single-arm open-label multicentre trial. *Heart* 111, 11-17. doi:10.1136/heartjnl-2024-324343

Wang, K., Xie, X., Hu, X., Wang, Z., Xia, J., and Wu, Q. (2024). Stearic acid alleviates aortic medial degeneration through maintaining mitochondrial dynamics homeostasis via inhibiting JNK/MAPK signaling. *iScience* 27, 110594. doi:10.1016/j.isci.2024.110594

Wang, X., Kuang, J., Li, X. T., Hu, X., Liu, Y. H., Hu, C. P., et al. (2025). Dimethyl fumarate is repurposed to ameliorate aortic aneurysm and dissection in mice. *Eur. J. Pharmacol.* 988, 177215. doi:10.1016/j.ejphar.2024.177215

Wu, S. K., Wang, L., Wang, F., and Zhang, J. (2024). Resveratrol improved mitochondrial biogenesis by activating SIRT1/PGC-1α signal pathway in SAP. *Sci. Rep.* 14, 26216. doi:10.1038/s41598-024-76825-9

Yu, X., Jiang, D., Wang, J., Wang, R., Chen, T., Wang, K., et al. (2019). Metformin prescription and aortic aneurysm: systematic review and meta-analysis. *Heart* 105, 1351-1357. doi:10.1136/heartjnl-2018-314639

Zhang, X., Zhang, Z., Wan, S., Qi, J., Hao, Y., An, P., et al. (2024). Ameliorative Effect of Coenzyme Q10 on Phenotypic Transformation in Human Smooth Muscle Cells with FBN1 Knockdown. *Int. J. Mol. Sci.* 25, doi:10.3390/ijms25052662
